# Drug discovery inspired by bioactive small molecules from nature

**DOI:** 10.1080/19768354.2022.2157480

**Published:** 2022-12-21

**Authors:** Seyun Kim, Seol-Wa Lim, Jiyeon Choi

**Affiliations:** Department of Biological Sciences, Korea Advanced Institute of Science and Technology (KAIST), Daejeon, Republic of Korea

**Keywords:** Natural product, drug discovery, metabolite, cell signaling, metabolomics

## Abstract

Natural products (NPs) have greatly contributed to the development of novel treatments for human diseases such as cancer, metabolic disorders, and infections. Compared to synthetic chemical compounds, primary and secondary metabolites from medicinal plants, fungi, microorganisms, and our bodies are promising resources with immense chemical diversity and favorable properties for drug development. In addition to the well-validated significance of secondary metabolites, endogenous small molecules derived from central metabolism and signaling events have shown great potential as drug candidates due to their unique metabolite-protein interactions. In this short review, we highlight the values of NPs, discuss recent scientific and technological advances including metabolomics tools, chemoproteomics approaches, and artificial intelligence-based computation platforms, and explore potential strategies to overcome the current challenges in NP-driven drug discovery.

## Introduction

Humans has long relied on natural products (NPs) to treat and manage various diseases including infection, cancer, and metabolic disorders (e.g. obesity and type 2 diabetes) (Koehn and Carter 2005; Baker et al. 2007; Cragg and Newman 2013; Atanasov et al. 2021). Historical records from Mesopotamia dating back to 2,600 BC clearly demonstrate that early civilizations were aware of the medicinal properties of approximately 1,000 plants. Traditional Asian medicine is also based on the knowledge obtained from thousands of years of disease management and treatment using NPs. Since the nineteenth century, the concept of rational drug discovery began to flourish with the successful isolation of analgesic chemicals from opium and many bioactive compounds such as quinine, nicotine, and rapamycin from various natural sources. Driven by the industrial need to maximize production yields and quality, natural compounds such as salicylic acid have been successfully synthesized through chemical procedures. The discovery of penicillin further broadened our interests by recognizing the potent activities of microbial NPs. Crude and semi-pure extracts from medicinal plants, animals, and microbes including fungi provided the best available medications in this early period. However, with the introduction and validation of the receptor theory of drug action in pharmacology, specific chemical compounds in crude NP extracts were identified as the primary factors mediating the biological and pharmacological properties of these extracts. Well-known examples of approved drugs derived from NPs include penicillin (antibacterial), morphine (analgesic), artemisinin (antimalarial), fingolimod (immunosuppressor), rapamycin (immunosuppressor, anticancer), and paclitaxel (anticancer). Small bioactive molecules from NPs are also widely used to improve biological activity and pharmaceutical properties, as demonstrated by the use of salicylic acid for aspirin production.

Our intense efforts to investigate NPs as sources of novel human therapeutics became fruitful between the 1970s and 1980s, leading to pharmaceutical development influenced by non-synthetic molecules. While classical and combinatorial chemical compound libraries are competitively emerging, mainly due to their favorable physicochemical properties and lower toxicity, NPs have been continuously considered an attractive source of compounds for drug discovery (Figure 2). Nearly 25% of new drugs approved worldwide in the past four decades are NPs and their derivatives, whereas another 25% are synthetic drugs with an NP pharmacophore or drugs that mimic the structure and properties of an NP (Newman and Cragg 2020). These novel drugs have been used to treat a wide variety of disorders including infectious (bacterial, fungal, parasitic, and viral), immunological, cardiovascular, neurological, inflammatory, and related diseases, as well as cancer.

Compared to synthetic small molecules, the most valuable feature of NPs as a resource for drug discovery is their structural scaffold complexity (Rodrigues et al. 2016; Yñigez-Gutierrez and Bachmann 2019; Lautié et al. 2020). All small molecules found in nature are metabolites, which are defined as intermediate or final products of metabolism (e.g. synthesis and breakdown of carbohydrates, proteins, fats, and nucleic acids) catalyzed by various arrays of cellular enzymes. Primary metabolites are the chemical compounds produced from central metabolic processes, thus contributing to the regulation of energy homeostasis, growth, and reproduction. Secondary metabolites derived from central metabolic pathways in plants and fungi are not required for homeostatic metabolic processes. However, they offer a wide array of new chemical structures, many of which have numerous biological and pharmacological properties against virtually every existing disease including cancer (Seca and Pinto 2018; Keller 2019). In this short review, we summarize how our knowledge of the biological activities of primary metabolites can be used for drug discovery. We then highlight the importance of secondary metabolite activities and finally focus on current/future strategies to harness their therapeutic properties and promote drug discovery and development.

## Emerging roles of primary metabolites as signaling factors

**Figure 1.**
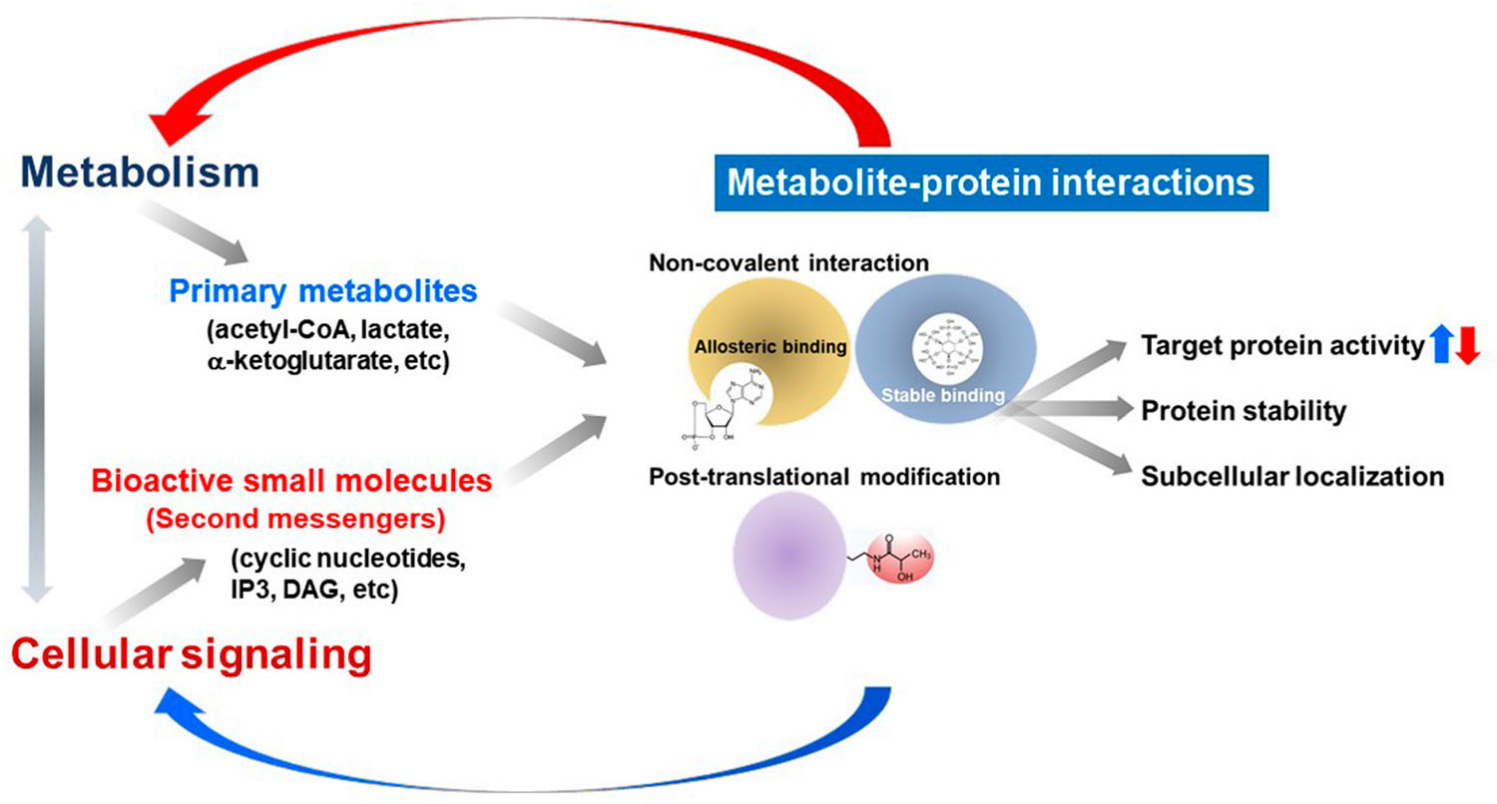
Biological actions of endogenous small molecules in the control of cellular signaling and metabolism via metabolite-protein interactions.

Primary metabolites have long been viewed as a simple fuel source for energy metabolism or as fundamental substrates required for the degradation and biosynthesis of macromolecules. However, several recent discoveries have demonstrated that certain primary metabolites can trigger or mediate potent biological activities (Figure 1) (Fang et al. 2001; Schreiber 2005; Keller et al. 2012; Shimazu et al. 2013; Lee et al. 2015; Mills et al. 2018; Bae et al. 2020; Gomes et al. 2020; Harayama and Shimizu 2020; Lee et al. 2020c; Martínez-Reyes and Chandel 2020; Shyer et al. 2020; Lee et al. 2021). Particularly, these metabolites can control cellular signaling pathways, which are the main targets for drug development (Li and Snyder 2011; Wang and Lei 2018; Milanesi et al. 2020). These findings suggest that our cellular and physiological systems have evolved to utilize specialized endogenous small molecules as primary or secondary signaling messengers to fine-tune various biological events. Therefore, identifying specific cellular metabolite-protein interactions provides insights into the development of synthetic drug molecules with enhanced properties such as efficacy and stability (Piazza et al. 2018). Endogenous small molecules can thus be classified depending on their modes of interaction (Figure 1).

First, small molecules in our body can act as ligands toward specific receptor molecules. In addition to the well-known involvement of hydrophobic steroid hormones in the activation of nuclear receptor proteins (Mangelsdorf et al. 1995), primary metabolites (e.g. adenosine, sphingosine-1-phosphate, free fatty acids, amino acid neurotransmitters) also stimulate key signaling pathways for cellular responses such as cell growth, differentiation, immunity, neuronal activation, and morphological changes by directly binding and stimulating their cognate cellular receptors (Schreiber 2005; Jacobson and Gao 2006; Traynelis et al. 2010; Blaho and Hla 2014; Milligan et al. 2017).

Furthermore, our metabolism produces second messenger molecules such as cyclic nucleotides, inositol polyphosphates, and bioactive lipids upon activation of target cells (Berridge and Irvine 1984; Conti 2000; Murad 2006; Newton et al. 2016). The production and degradation of these second messengers are tightly regulated by the dynamic changes of cellular and physiological programs such as differentiation, cellular death, development, and tissue regeneration. Recent studies on cellular nutrient and energy sensing continue to uncover unexpected metabolite-protein interactions (e.g. leucine-sestrin, inositol pyrophosphate-Akt) (Alvarez et al. 2010; Chakraborty et al. 2010; Lee et al. 2016; Lee et al. 2020c; Wolfson et al. 2016; Li et al. 2017). Moreover, druggable targets can be further developed based on our knowledge of the allosteric interaction between endogenous small molecules and their effector proteins. Some primary metabolites are also known to mediate stable interactions with target proteins, suggesting their pivotal roles in coordinating protein stability. For example, inositol polyphosphate is known as an essential factor to mediate capsid formation for HIV (human immunodeficiency virus) viral particle assembly, suggesting that IP6 (inositol hexakisphosphate) metabolism is a promising target for the development of new anti-HIV therapeutics (Dick et al. 2018). The stabilization of the molecular complexes (e.g. RNA editing enzyme ADAR2, Integrator) by inositol polyphosphates is another example demonstrating the significance of stable metabolite-protein interactions for the development of novel therapeutics (Macbeth et al. 2005; Lin et al. 2022).

Besides the above-mentioned non-covalent metabolite-protein interactions of cellular metabolites, some metabolic biomolecules can also lead to various post-translational modifications (PTMs) (Figlia et al. 2020). In addition to ATP, which is an essential substrate for protein phosphorylation, many other metabolites can also modify and modulate target protein functions. For example, nitric oxide (NO), a product derived from arginine metabolism, is the primary source for protein S-nitrosylation, a process that leads to changes in the activities, subcellular localization, and stability of target proteins (Jaffrey et al. 2001; Stomberski et al. 2019). S-adenosyl methionine, acetyl-CoA, and other metabolites (e.g. alpha-ketoglutarate) can indeed control many signaling pathways and mediate epigenetic changes through unique PTMs (e.g. methylation, acetylation, succinylation) (Kaelin and McKnight 2013). The identification of other PTMs and their responsible metabolites could provide specific molecular targets and metabolism for novel drug discovery and development.

Recent advances in the characterization of primary metabolites and endogenous small molecules as signaling factors have greatly expanded our understanding of the vast potential of NPs as sources of therapeutic compounds. Fundamental structural and biological information on the specific covalent and/or non-covalent interactions among bioactive primary metabolites and target proteins provide a valuable resource for the development of efficient small molecules and therapeutic compounds based on known druggable targets. In addition to targeting metabolite-protein interactions, approaches to modulate selective metabolites will also become an important strategy to control serious diseases such as cancer (Wang and Lei 2018; Milanesi et al. 2020; Stine et al. 2022). As exemplified by recent studies, increasing the levels of toxic metabolites can be selectively and efficiently kill tumor cells (Kim et al. 2015; Lee et al. 2020b). In summary, the primary metabolites in our cells and tissues are less structurally complex compared to secondary metabolites from other organisms, but endogenous small molecules and their control of cellular signaling should be further elucidated and applied for the development of next-generation drugs.

## Pharmacologically-active secondary metabolites as major resources for the discovery of potent drugs

Many medicinal NPs derived from plants, fungi, and microorganisms have long been used to alleviate and cure a wide range of medical conditions such as infectious diseases, inflammatory reactions, obesity/diabetes, cardiovascular disease, and cancer, as well as psychiatric disorders (Dias et al. 2012; Atanasov et al. 2021). Our information on these potent effects of extracts and/or single compounds derived from NPs have been continuously updated (Tables 1 and 2). The potent activities of these NPs largely originate from their secondary metabolites, which are organic compounds produced through the modification of primary metabolites. Unlike primary metabolites, secondary metabolites and related metabolic reactions are involved in ecological functions (e.g. pathogen sensing and defense mechanisms) (Li et al. 2020).

**Table 1.** Selected examples of bioactive NP extracts.

Sources	In vitro & biological effects (References)
*Phloms lanata*	Anti-oxidative effects, T cell activity control (Couladis et al. 2003; Karali et al. 2016)
Microalgae *Euglena tuba*	Anti-tumor effects (Shanab et al. 2012; Gupta et al. 2022)
*Holothuria atra*	Anti-tumor effects (Nursid et al. 2019; Cui et al. 2022; Nugroho et al. 2022)
*Polyalthia evecta*	Anti-tumor effects (Machana et al. 2012)
*Solieria robusta*	Anti-tumor effects (Yen et al. 2014)
*Tagetes erecta*	Anti-oxidative, anti-tumor effects (Burlec et al. 2021; Cui et al. 2021)
*Sorbus commixta Sorbus acuparia, Sorbus caucasica*	Anti-tumor, anti-bacterial effects (Park et al. 2017)
*Populus tremuloides*	Anti-microbial effects (Turumtay et al. 2017; St-Pierre et al. 2018)
*Jatropha gossypiifolia*	Antmicrobial, anti-inflammatory, antidiarrheal, antihypertensive, anti-tumor effects (Félix-Silva et al. 2014)
*Terminalia macroptera*	Anti-malarial, anti-inflammatory, anti-psychotic effects (Pham et al. 2014; Ior et al. 2021)
*Onopordum acanthium*	Anti-inflammatory, anti-tumor, cardiotonic effects (Lajter et al. 2015; Robertovna et al. 2019)
Brown algae (e.g. *Ascophyllum nodosum*)	Anti-tumor, anti-diabetic effects (Austin et al. 2018; Gabbia and Martin 2020)
*Allium sativum*	Anti-oxidative, anti-tumor, lipid-lowering effects, gastrointestinal motility control (Kimura et al. 2017; Moon et al. 2022)
*Mangifera indica*	Anti-oxidative, anti-inflammatory, anti-diabetic, immunomodulatory, anti-tumor effects (Noratto et al. 2010; Jung et al. 2022)
*Agrimonia pilosa*	Anti-oxidative, anti-inflammatory, anti-tumor, analgesic effects (Feng et al. 2022; Wen et al. 2022)
*Clerodendrum trichotomum*	Anti-inflammatory, anti-microbial, anti-viral, anti-tumor, anti-diabetic effects (Kim et al. 2009; Jang et al. 2021)
*Geranium thunbergii*	Anti-tumor, anti-obesity effects (Sung et al. 2011; Choi et al. 2015; Lee et al. 2020a)
Colored corn (*Zea mays L.)*	Ant-bacterial, anti-oxidative, anti-inflammatory, anti-diabetic effects, neuroprotection (Colombo et al. 2021; Kim et al. 2021b; No et al. 2021)

**Table 2.** Selected list of bioactive secondary metabolites derived from plant NPs.

Metabolites	Sources	In vitro & biological effects (References)
Avenanthramide	*Avena sativa*	Anti-oxidative, anti-inflammatory actions (Collins 1989; Koenig et al. 2016; Lim and Kang 2020)
Apigenin	Fruits, vegetables	Anti-oxidative, anti-inflammatory, anti-tumor effects (Bao et al. 2013; Rahmani et al. 2022)
Baicalein	*Scutellaria*species	Anti-oxidative, anti-bacterial, anti-inflammatory, anti-tumor effects (Chandrashekar and Pandi 2022)
Betulinic acid	Plants (Betula sp.)	Anti-oxidative, anti-inflammatory, hepatoprotective, anti-tumor effects (Ríos and Máñez 2018; Park et al. 2021)
Chloroquine	*Cinchona*sp.	Anti-malarial, anti-tumor, immune suppressive, autophagy-inhibitory effects (Zhou et al. 2020; Sharma et al. 2021)
Chrysin	Honey, vegetables, (*Pelargonium crispum*, *Passiflora incarnate*, etc.)	Anti-oxidative, anti-inflammatory, anti-tumor, anti-nociceptive effects, neuroprotection (Mani and Natesan 2018; Hong et al. 2020; Stompor-Gorący et al. 2021)
Cynarin	*Cynara cardunculus*	Anti-oxidative, antihypertensive, anti-inflammatory, anti-atherosclerotic, anti-HIV, anti-tumor, cholesterol-lowering effects (Topal et al. 2016; Hakkou et al. 2017; Kim et al. 2022a)
Decursin	*Angelica gigas*	Anti-inflammatory, anti-tumor, anti-angiogenic effects (Yim et al. 2005; Shehzad et al. 2018; Ahmed et al. 2020)
Ginsenoside	*Panax ginseng*	Anti-oxidative, anti-tumor, anti-inflammatory, anti-aging, cognitive improvement effects (Ratan et al. 2021; Hou et al. 2022)
Hesperidin	Vegetables	Anti-oxidative, anti-tumor, anti-inflammatory effects, gastrointestinal motility control (Garg et al. 2001; Hwang et al. 2020)
Honokiol	*Magnolia officinalis*	Anti-oxidative, anti-tumor, anti-inflammatory, anti-diabetic effects, protective actions in various organs (e.g. liver, brain) (Park et al. 2020; Rauf et al. 2021)
Kazinol	*Broussonetia kazinoki*	Anti-tumor, autophagy-promoting effects (Kim et al. 2015; Lee et al. 2022)
Lapachone	*Tabebuia avellanedae*	Anti-oxidative, anti-tumor, anti-inflammatory, anti-aging (Gomes et al. 2021)
Luteolin	Aromatic flowering plants, vegetables, *Salvia tomentosa, Aiphanes aculeate*	Anti-oxidative, anti-inflammatory, anti-atherosclerotic, anti-thrombogenic effects, neuroprotection, cardioprotection, mood control (Mahdiani et al. 2022; Sur and Lee 2022)
α-Mangostin	*Garcinia mangostana*	Anti-oxidative, anti-tumor, anti-inflammatory, anti-diabetic, anti-obesity, hepatoprotection, cardioprotection (Zhang et al. 2017; John et al. 2022)
Quercetin	Fruits, vegetables	Anti-oxidative, anti-tumor, anti-inflammatory, anti-diabetic, anti-aging effects (Deepika 2022; Jan et al. 2022)
Rosmarinic acid	Boraginaceae family and the subfamily Nepetoideae of the Lamiaceae family	Anti-oxidative, anti-tumor, anti-inflammatory, anti-depressive effects (Moore et al. 2016; Luo et al. 2020; Jeong et al. 2021)
Resveratrol	Red grapes, blueberries, and many food products (soy, nuts, etc)	Anti-oxidative, anti-tumor, anti-inflammatory, anti-diabetic, anti-aging, autophagy-promoting effects (Fu et al. 2021; Zhang et al. 2021)
Plumbagin	*Plumbago zeylanica*	Anti-oxidative, anti-tumor, anti-inflammatory, anti-bacterial effects (Hwang et al. 2015; Cai et al.^2020^)
Rutin	Fruits (e.g. oranges, lemons, grapes)	Anti-oxidative, anti-tumor, anti-inflammatory effects, hepatoprotection (Enogieru et al. 2018; Choi et al. 2021)
Tannic acid	Fruits, vegetables	Anti-oxidative, anti-tumor, anti-inflammatory effects, neuroprotection, cardioprotection (Luduvico et al. 2020; Jing et al. 2022; Kim et al. 2022b)

Structural diversity and complexity are the most important features of secondary metabolites as an essential resource for drug in competition with synthetic chemical libraries (Hong 2011; Lautié et al. 2020). Approximately 40% of the chemical scaffolds from NPs are not found in commercial synthetic compounds (Henkel et al. 1999). Furthermore, 83% of the ring scaffolds in NPs are not present in synthetic molecules (Hert et al. 2009). Complex molecules can more readily interact with a greater variety of chemical structures and modifications, suggesting a superior potential for secondary metabolites to complement the spatial characteristics of target proteins. Although many secondary metabolites do not fit into drug-likeness standards such as Lipinski's ‘rule of five’ (e.g. logP ≤ 5, molecular weight ≤ 500 Da, number of hydrogen bond acceptors ≤ 10), they exhibit more favorable metabolic and pharmacokinetic properties such as absorption, distribution, metabolism, and excretion/toxicity than synthetic molecules (Müller-Kuhrt 2003; Atanasov et al. 2021).

**Figure 2.**
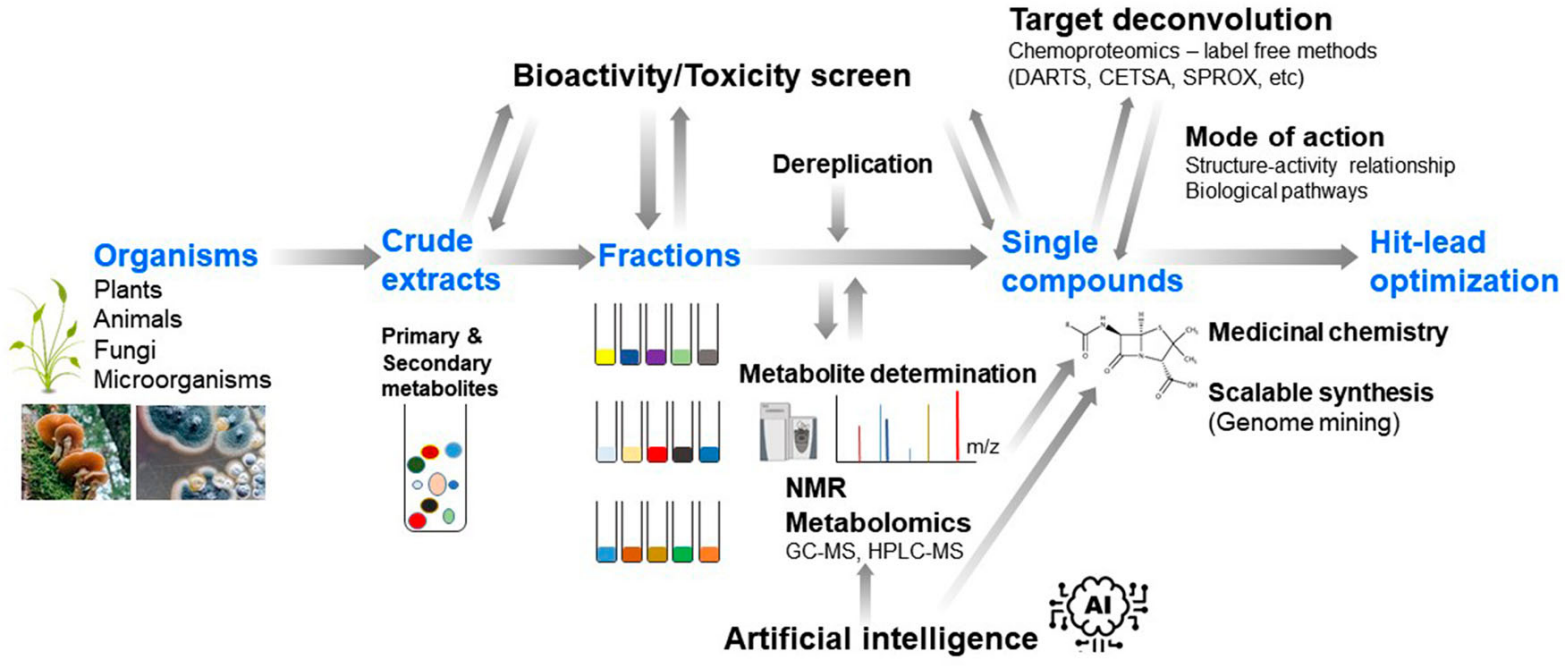
Strategy of secondary metabolites-inspired drug discovery.

The journey from the identification of promising bioactive secondary metabolites to drug discovery indeed entails a series of demanding processes (Figure 2). The therapeutic potential of secondary metabolites depends on the quality and quantity of the bioactive chemicals in medicinal organisms, which in turn is influenced by various environmental factors (e.g. growth conditions, age, climate changes). The purification of bioactive metabolites involves various strategies such as combinatorial chemistry, isolation assays, and efficacy-based high-quality fractionation. It is also critical to avoid the replication of previous efforts by correctly identifying known compounds. The determination of *de novo* structure of novel compounds has greatly benefited from recent advances in spectroscopic techniques such as high-resolution nuclear magnetic resonance (NMR) technologies. When the biological activity profile of a therapeutic candidate meets the optimal criteria for potency and selectivity, structure–activity relationship (SAR) studies are then conducted and large-scale purification processes are developed. Once synthetic modification methods become feasible, hit-to-lead optimization is further accelerated by conventional medicinal chemistry approaches.

## Strategies for accelerating natural metabolite-driven drug discovery

To continue the long and successful history of NPs in drug discovery and their unique structural diversity, several challenges must be overcome (Koehn and Carter 2005; Lam 2007; Atanasov et al. 2021). NP-derived lead compounds typically exhibit low solubility or chemical instability, which impedes further drug development. Many NPs also exhibit high molecular weight and complex structures, which often results in poor absorption and complicates the development of oral formulations. Although naturally active substances usually make well-qualified lead compounds, many of them can hardly fulfill the criteria for druggability. Therefore, the most important step during NP-based drug discovery is the efficient and accurate selection of natural sources for the extraction and isolation of bioactive metabolites with desired biological activities and structural properties (Figure 2).

Technical advancements in the field of metabolomics — the sensitive, unbiased, and high-throughput study of complex metabolites — have enabled the characterization and quantification of bioactive metabolites from complex mixtures derived from NPs (Liu and Locasale 2017). Metabolomics can thus be widely applied to the analyses of pharmaceutically relevant NP resources, as well as for the discovery of bioactive metabolites (Wishart 2016; Wolfender et al. 2019; Stuart et al. 2020). Coupling metabolomics with NMR further facilitates the acquisition of structural information of potent metabolites, which saves a substantial amount of time and labor when extracting or isolating metabolites (Lin et al. 2008; Gathungu et al. 2020). Efforts have been recently made to establish a comprehensive experimental tandem mass spectrometry (MS/MS) database of NPs. The Global Natural Products Social (GNPS) molecular networking platform contains thousands of MS datasets from NP extracts (Wang et al. 2016). Moreover, this platform clusters structurally related metabolites and provides insights regarding their relationships. In addition to the GNPS platform, the METLIN (Guijas et al. 2018) and CSI:FingerID (Dührkop et al. 2015) databases provide useful information to expedite metabolite identification by combining fragmentation tree computation and machine learning.

The subsequent process of identifying the molecular targets of bioactive NP-derived hits, which is also known as ‘target deconvolution,’ is essential for underpinning the mechanisms of drug action, as well as for the application of the identified hits to fully elucidate the biological processes modulated by a drug candidate (Terstappen et al. 2007). Recent advancements in chemical proteomics have led to the development of efficient and sensitive methods analyzing the proteins that the bioactive metabolite of interest binds. Based on the assumption that a molecule binding to a protein target alters the target’s stability, two major methods have been widely applied. Drug affinity-responsive target stability (DARTS) assesses the changes in the stability of a protein to proteolysis upon binding with the ligand (Lomenick et al. 2009). The cellular thermal shift assay (CETSA) and the stability of proteins from rates of oxidation (SPROX) rely on the thermal stabilization of a protein bound to a ligand (Molina et al. 2013; Strickland et al. 2013). Label-free metabolites of interest can be used in these platforms, thus foregoing the need for laborious chemical modifications such as biotinylation. Combined with MS proteomics, the CETSA-MS and DARTS-MS platforms become more powerful, thus enabling the acquisition of protein–ligand interactome data, as well as the accompanying physiological changes in biological samples such as cell lysates, intact cells, as well as tissues (Savitski et al. 2014; Pai et al. 2015).

Small molecule drug candidates derived from NPs can be applied by conjugating them with other bioactive molecules, thus expanding the use of NP metabolites for the development of novel, bifunctional, and more effective drugs for disease treatment. The immense range of bifunctional conjugates used for the development of NP hybrid drugs includes antibody–drug conjugates and aptamer drug conjugates, PROteolysis TArgeting Chimeras (PROTAC), and AUTOphagy-TArgeting Chimeras (AUTOTAC) (Yoon et al. 2017; Newman 2021; He et al. 2022; Ji et al. 2022). For example, the PROTAC approach is based on the development of bifunctional hybrid molecules comprised of a ligand for an E3 ligase and a ligand for the target protein joined by a linker, thus leading to the ubiquitination of the target protein and proteasomal degradation (Nalawansha and Crews 2020; Li and Crews 2022). For example, the PROTAC approach, which is based on the NP apigenin (i.e. a low estrogenic flavonoid with anticancer activity), was developed to specifically target the aryl hydrocarbon receptor for degradation (Puppala et al. 2008). Wogonin-based PROTACs were also used for the synthesis of CDK9-targeting PROTACs capable of selectively degrading CDK9 (Bian et al. 2018). Since the first study with PROTACs was performed with a natural polyketide ovalicin-derived molecule (Sakamoto et al. 2001), the use of NP-mediated target protein degradation has emerged as a promising strategy to treat diseases such as cancer and metabolic disease (Li et al. 2022).

Artificial intelligence (AI) has garnered increasing attention in various academic fields as well as industrial decision-making and processing applications because it allows for fast and efficient analysis and reduces human errors. Therefore, AI has recently been applied in drug discovery to analyze molecular properties, identify synthetic routes, and predict bioactive metabolites. By using various machine learning algorithms coupled with cloud computing technologies, big data accumulated from drug discovery and development can be processed to facilitate the identification of therapeutic candidates. For example, machine learning software (e.g. ACD/structure elucidator, Mestrelab Mnova) has been used for structure determination and dereplication (Claridge 2009; Elyashberg and Williams 2021). An AI-based structure prediction tool (DP4-AI) has been also developed to predict metabolite structures as well as MS2DeepScore, a machine learning-based mass spectral similarity-predicting algorithm, to identify metabolites based on clustering analysis (Howarth et al. 2020; Huber et al. 2021). Machine learning can also be used to identify drug targets. For example, BANDIT, a Bayesian machine-learning algorithm, is used to integrate multiple data types and predict the targets of nearly 4,000 compounds with a 90% accuracy (Madhukar et al. 2019). Other AI platforms include DEcRyPT (Drug–Target Relationship Predictor) (Rodrigues et al. 2018), SuperPred (Gallo et al. 2022), and NPClassifier (Kim et al. 2021a). The integration and curation of different forms of NP-derived databases (taxonomic, structural, genomic, transcriptomic, proteomic, and metabolomics databases) should be systematically pursued to overcome the common drawbacks of AI-powered technology for drug discovery. In turn, this approach is highly expected to reduce errors and increase predictability.

## Conclusions

The quest for the discovery of new drugs derived from natural metabolites has led to many breakthroughs and achievements (e.g. taxol, artemisinin, rapamycin, and penicillin). In addition to the discovery of several potent bioactive secondary metabolites, recent findings on the signaling activities of endogenous primary metabolites have greatly contributed to the identification of novel metabolite-protein interactions (Figure 1), which are critical for disease control. Recent technological improvements and systems biology approaches coupled with the application of available omics technologies and AI-powered computational strategies will potentially pave the way for the discovery of new NP-derived drug candidates (Figure 2). In turn, this strategic integration of various technologies enables the design of a new generation of first- and best-in-class drugs. Less than 1% of Earth’s vast biodiversity has been investigated as a potential source of drug candidates. However, the discovery of novel therapeutic compounds is being threatened by the massive destruction of ecosystems (e.g. deforestation) and the consequent loss of species diversity and habitats. Therefore, promoting NP research through the construction of metabolite databases and the use of integrative drug discovery platforms could develop new and more effective NP-based therapeutics, sooner than expected.
